# Harnessing the Power of Nanocarriers to Exploit the Tumor Microenvironment for Enhanced Cancer Therapy

**DOI:** 10.3390/ph18050746

**Published:** 2025-05-19

**Authors:** Bandar Aldhubiab, Rashed M. Almuqbil, Anroop B. Nair

**Affiliations:** Department of Pharmaceutical Sciences, College of Clinical Pharmacy, King Faisal University, Al-Ahsa 31982, Saudi Arabia; ralmuqbil@kfu.edu.sa (R.M.A.); anair@kfu.edu.sa (A.B.N.)

**Keywords:** tumor microenvironment, drug resistance, nanocarriers, enhanced pharmacokinetics, TME remodeling

## Abstract

The tumor microenvironment (TME) has a major role in malignancy and its complex nature can mediate tumor survival, metastasis, immune evasion, and drug resistance. Thus, reprogramming or regulating the immunosuppressive TME has a significant contribution to make in cancer therapy. Targeting TME with nanocarriers (NCs) has been widely used to directly deliver anticancer drugs to control TME, which has revealed auspicious outcomes. TME can be reprogrammed by using a range of NCs to regulate immunosuppressive factors and activate immunostimulatory cells. Moreover, TME can be ameliorated via regulating the redox environment, oxygen content, and pH value of the tumor site. NCs have the capacity to provide site-specific delivery of therapeutic agents, controlled release, enhanced solubility and stability, decreased toxicities, and enhanced pharmacokinetics as well as biodistribution. Numerous NCs have demonstrated their potential by inducing distinct anticancer mechanisms by delivering a range of anticancer drugs in various preclinical studies, including metal NCs, liposomal NCs, solid lipid NCs, micelles, nanoemulsions, polymer-based NCs, dendrimers, nanoclays, nanocrystals, and many more. Some of them have already received US Food and Drug Administration approval, and some have entered different clinical phases. However, there are several challenges in NC-mediated TME targeting, including scale-up of NC-based cancer therapy, rapid clearance of NCs by the mononuclear phagocyte system, and TME heterogeneity. In order to harness the full potential of NCs in tumor treatment, there are several factors that need to be carefully studied, including optimization of drug loading into NCs, NC-associated immunogenicity, and biocompatibility for the successful translation of NC-based anticancer therapies into clinical practice. In this review, a range of NCs and their applications in drug delivery to remodel TME for cancer therapy are extensively discussed. Moreover, findings from numerous preclinical and clinical studies with these NCs are also highlighted.

## 1. Introduction

Cancer is a leading cause of death worldwide and there is a continuous increase in the number of cancer patients despite advancements in anticancer therapies [1,2]. It has been reported that approximately one in five men and one in six women will develop cancer in their lifetime, while around one in 12 women and one in nine men will die from cancer [3]. The major cause of the poor efficacy of therapies is the low targeting ratio of therapies, which can further damage healthy normal tissues [4,5]. Thus, there is an urgent and unmet need for more site-specific delivery of therapies to tumor sites [6]. Cancer involves a complex ecosystem including a multitude of non-cancerous cells and tumor cells, which are embedded in an altered extracellular matrix [7]. The tumor microenvironment (TME) is composed of chemokines, vasculature, extracellular matrix (ECM), and a range of cells, including lymphocytes, inflammatory cells, endothelial cells, fibroblasts, and immune cells [8]. The immune cells can mediate both adaptive and innate immune responses. In TME, the innate immune cells, such as dendritic cells (DCs) and macrophages, are both anti- and pro-tumorigenic, contingent on multifaceted cross-talk and different chemokines. Innate immune cell-activated adaptive immune systems can specifically target and attack tumor cells, which is found to be most effective for the eradication of tumors. Activated fibroblasts present in tumors are known as cancer-associated fibroblasts (CAFs), which make up the structure of the microenvironment via generating a large proportion of the ECM in TME and have a significant impact on tumor progression and tumor therapy [9].

A feature of TME is abnormal tumor vasculature along with abnormal structural and functional dynamics, which can further mediate hypoxia. The hypoxic condition in TME can restrict therapeutic effects, promote tumor progression, and alter activities of the normal microenvironment. Thus, the interactions between TME components and tumor cells can influence therapeutic effects, tumor progression, and metastasis. TME can greatly affect a drug’s penetration as well as its functions and is linked with low response rates and drug resistance [10,11,12]. In addition, TME can lead to poor treatment outcomes in the case of cancer immunotherapy, targeted therapy, and chemotherapy [13,14,15]. An immunosuppressive TME can support the development and occurrence of tumors, which can result in immune escape of tumor cells [16]. There is a growing interest in modulating TME to ameliorate the efficacy of cancer therapies [17]. Therefore, it is necessary to modulate TME by targeting extracellular ligand–receptor interactions and downstream signaling cascades, which can further ameliorate therapeutic effectiveness and attain durable responses [18,19]. The effectiveness of conventional cancer treatments is limited to a great extent due to TME-associated immunosuppressive mechanisms. Various immune escape processes have been discovered. In addition to TME-associated processes, immune escape processes involve various epigenetic, genetic, metabolic, and humoral factors within TME [20].

In the past few decades, nanocarriers (NCs) have been extensively studied, since they have demonstrated great therapeutic promise as drug delivery systems. NCs are colloidal drug carrier systems composed of nanosized particles with a size <500 nm. NCs are used for specific spatial placement and triggered drug release within the target cancer cells only [21,22]. Because of the high surface-area-to-volume ratio, NCs have the capacity to modify the basic features and bioactivity of drugs. Such features include site-specific delivery of therapeutic agents, controlled release, enhanced solubility and stability, decreased toxicities, and enhanced pharmacokinetics and biodistribution [23,24]. The physicochemical properties of NCs can also be modified by altering their surface properties (such as attachment of targeting moieties, PEGylation or other coating, functional groups, surface charge), shapes (cube, rod, or sphere), sizes (large or small), and compositions (inorganic, organic, or hybrid) [25,26]. NCs are mainly used as drug delivery systems for effective drug delivery along with minimum side-effects [22]. Nanocarrier-based drug delivery systems have greatly enabled efficient delivery of various antineoplastic agents into tumor sites via modulating TME pathophysiology, thus markedly enhanced the therapeutic outcomes for a range of cancer types [22,27,28,29,30]. In recent times, there has been a growing interest in stimuli-responsive nanocarriers that can be functionalized to deliver, release, and activate cargos in targeted areas, including TME or the intracellular spaces of cancer cells, in response to external or internal stimuli, including enzymes and pH [31,32,33,34,35]. Interestingly, pH-responsive polymer NCs carrying anticancer drugs can alter their properties or structures with pH reduction in TME, which provides precise targeted tumor therapy [36].

In addition, NCs with modulators, including multifunctional platforms, can efficiently suppress distal metastasis, eradicate primary cancer, and avert the recurrence of cancers [37,38]. NCs primarily modulate TME through different mechanisms, including improving the TME microenvironment [39], reversing immunosuppressive cells [40], activating disabled immune cells [41], and mediating the immunogenicity of cancer antigens [42]. In this article, a range of NCs and their applications in drug delivery to remodel TME for cancer therapy are extensively discussed. Moreover, the findings from numerous preclinical and clinical studies with these NCs are also highlighted. This review uniquely provides an updated and comprehensive discussion on how NCs can be strategically engineered and used to target and modulate TME for enhanced therapeutic outcomes. This review also provides an important discussion regarding deeper understanding of TME biology, limitations of various NCs, their manufacture as well as translational challenges, and approaches for bridging the gap between preclinical research and real-world applications.

## 2. The Complexity of the Tumor Microenvironment (TME) and Its Impact on Cancer

**Figure 1:**
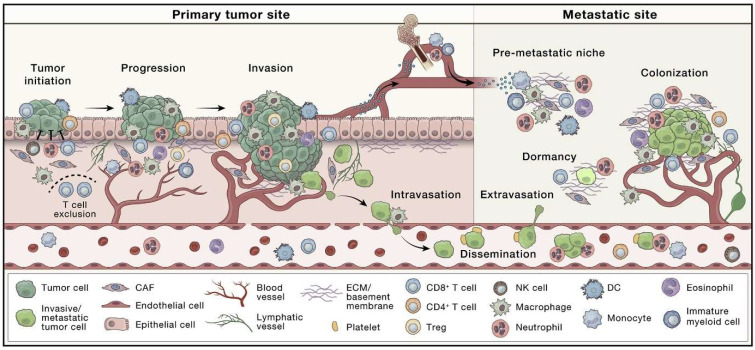
The role of tumor microenvironment (TME) components in the regulation of tumor growth and metastasis. Reproduced with permission from Elsevier, Reference [7]. TME involves a range of cell types, including microglia, fibroblasts, endothelial cells, pericytes, immune cells, and various other tissue-resident cell types. Interactions between the structural and cellular components of TME mediate cancer cells becoming invasive and spreading beyond the place where a tumor started to distant regions of the body, via a multi-stage and complex metastatic pathway. Growth-mediating and immunosuppressive properties are exhibited by tumor-associated macrophages (TAMs), exosomes that elevate the migratory capacity of cancer cells are generated by mesenchymal stem cells, and cancer-associated fibroblasts rearrange TME that enables metastasis of cancer cells. In addition, hypoxia at the primary tumor triggers cancer cells to genetically and/or epigenetically acclimatize to endure as well as metastasize. Cancer cells in the circulation encounter cytokines, immune cells, and platelets in the blood TME that mediate their transit and survival [7,60].

The complex nature of cancer becomes apparent after microscopic examination of a solid tumor, which reveals that TME is a highly complex ecosystem that surrounds a tumor [43]. A growing volume of evidence has demonstrated that TME plays significant roles in tumor survival, metastasis, immune evasion, and drug resistance [44,45,46,47]. TME involves a range of cell types, including microglia, fibroblasts, endothelial cells, pericytes, immune cells, and various other tissue-resident cell types (Figure 1). These cells interact with tumor cells to form a network of cell-to-matrix and cell-to-cell interactions, known as TME [48]. Concurrently, the redox condition, the extent of oxygen enrichment, and the pH value of tumor cells are markedly different from normal cells because of the impact of the proliferation of tumor cells [16,49]. It has been demonstrated that several interactions between tumor cells and their adjacent TME are crucial to comprehend the different underlying processes of tumor growth and the development of metastasis [50]. Further progress, carcinogenesis, and loss of tissue integrity take place because of the reciprocal interactions between tumor cells and the cellular as well as non-cellular (ECM) components of TME [51,52].

Interestingly, interactions between ECM, reactive non-neoplastic cells, and genetically altered tumor cells play a crucial role in most of the steps of tumorigenesis, including apoptosis, neovascularization, development of metastasis, invasion, cancer heterogeneity, migration, clonal evolution, chemotherapeutic drug resistance, and epithelial-mesenchymal-transition [53,54]. TME has a major role in malignancy; thus, numerous studies have focused on this research area [55,56]. Better knowledge regarding the mechanisms through which TME influences cancer advancement is likely to reveal novel targets that will be useful for the isolation of cancer cells and also for cancer treatment. This could be attained via interfering with the complex crosstalk between host cells, cancer cells, and their surrounding ECM [52]. TME recapitulation is a major challenge in experimental cancer model development. A suitable tool needs to be established for the development of personalized cancer therapies; thus, it is important to preserve the important features of the original tumors. In recent times, progress on three-dimensional platforms through the usage of microfluidic lab-on-a-chip devices has indicated great potential in better simulating the biology and functions of TME and in bridging the translational gap between clinical and preclinical settings [57,58,59].

## 3. Applications of Nanocarrier-Based Cancer Therapy Through Targeting the Tumor Microenvironment

**Figure 2:**
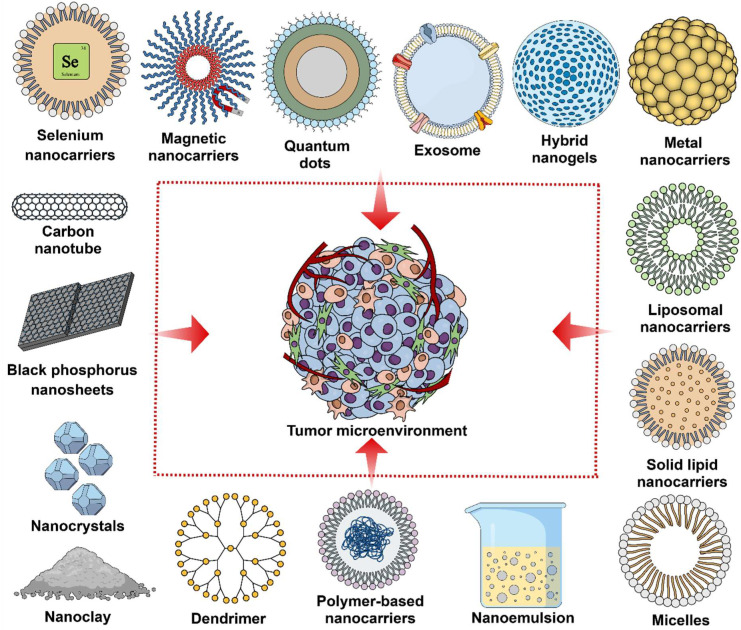
Potential nanocarriers to modulate the tumor microenvironment for enhanced cancer therapy.

The complex nature of TME restricts anticancer therapy [61]. TME barriers need to be overcome to achieve deep transfer of therapeutics and to enhance treatment outcomes. Thus, reprogramming or regulating immunosuppressive TME has a significant contribution in cancer therapy. Targeting TME with NCs (Figure 2) has been widely used to directly deliver anticancer drugs to control TME, which has revealed auspicious outcomes [62]. TME can be reprogrammed by using a range of NCs to regulate immunosuppressive factors, activate immunostimulatory cells, and destroy ECM. Moreover, TME can be ameliorated via regulating the redox environment, oxygen contents, and pH value of the tumor sites [16].

### 3.1. Metal Nanocarriers

Gold NCs are considered a promising drug delivery system in cancer immunotherapy because of their features, including ease of controlling shape and size, tunable surface chemistry, and biocompatibility [63]. These NCs have already demonstrated their potential in enhancing the differentiation of macrophages into dendritic-like cells to induce the proliferation of T-cells and mediate the release of cytokines [64]. Gold NCs were also found to be effective as adjuvants for increasing the generation of antibodies [65]. In cancer therapy, the efficacy of gold NCs in the modulation of TME has also been reported [66,67]. CpG-oligodeoxynucleotides have been conjugated to hollow gold NCs to improve their functions and cellular uptake in the stimulation of immune responses, such as inducing the secretion of TNF-α [68]. CAFs are important TME components that control cancer metabolism, invasion, metastasis, migration, growth, immunity, angiogenesis, cancer metabolism, and therapeutic resistance [69,70]. In a study, Zhang et al. [71] reported that TME and ovarian cancer cells can cause activation of ovarian CAFs, while 20 nm gold NCs suppress the activation, as confirmed through alterations in molecular markers, cell morphology, and migration. In addition, gold NCs altered the extent of several fibroblast inactivation or activation proteins, including thrombospondin-1, urokinase-type plasminogen activator, platelet-derived growth factor, and transforming growth factor-beta 1. Therefore, gold NCs can serve as an effective tool to facilitate understanding regarding multicellular communications that are present in TME and to develop approaches to disturb this communication [71].

On the other hand, silica NCs have also been widely used in the delivery of genes and drugs, specific targeting of cancer, and biomedicine for imaging [72,73]. In addition, mesoporous silica NCs have been used to deliver antigens and have also played a role as a vaccine delivery platform to enhance cell- and humoral-mediated immune responses, while lacking toxicity and exhibiting high biocompatibility [74]. Hollow mesoporous silica NCs are biodegradable and have the capacity for TME remodeling in the case of cancer immunotherapy [75].

### 3.2. Liposomal Nanocarriers

Liposomes are made up of amphiphilic membranes of synthetic or natural lipids. Liposomes also have the capacity to load hydrophobic agents in the lipid bilayer and hydrophilic drugs in the water core, which demonstrates their flexibility and potential as delivery vehicles [76,77]. Liposomal NCs also have the capacity to ameliorate the pharmacodynamics and bioavailability of poorly soluble drugs. In addition, the advantages of liposomal NCs include low immunogenicity, high biocompatibility, low toxicity, cell-like membrane, and capacity to provide protection to drugs from hydrolysis as well as prolongation of their biological half-life [78]. Liposomal NCs also have the capacity to encapsulate either hydrophilic or hydrophobic drugs and control their release [79,80]. However, there are several limitations of liposomal NCs, including manufacturing challenges, lack of targeting strategies, and slow overall shift of approved therapies into clinics [81].

TME can markedly contribute to inducing the metastasis and stemness properties of cancer cells. In a study, Guo et al. [82] prepared novel CD44-targeted liposomal NCs loaded with anti-IL6R antibodies, which have the capacity to selectively target TME of CD44^+^ breast cancer cells in luminal and triple-negative breast cancer (TNBC) mouse models. The NCs showed specific and enhanced tumor-targeting effectiveness with substantial anti-metastatic activities in syngeneic BALB/c mouse models containing 4T1 cells, as in syngeneic MMTV-PyMT mouse models. It also moderated TME and suppressed the IL6R-Stat3 signaling cascade, as characterized through the decreased expression of various genes encoding CD206, MMP-9, VEGFA, Sox2, and Stat3 in breast tissues. Collectively, the NCs significantly suppressed the metastasis of breast cancer in various mouse models for breast cancer [82].

Ultrasound-activated sonodynamic therapy (SDT) is a safe cancer therapeutic approach with a deep tissue penetration action. Concurrent integration of effective therapeutic drugs, controllable drug delivery vehicles, and ultrasound imaging contrast agents is a promising approach for cancer therapy [83]. Lin et al. [84] developed a 2,2′-azobis [2-(2-imidazolin-2-yl)propane]dihydrochloride-loaded liposome that can concurrently produce gas bubbles as well as a high level of reactive oxygen species (ROS) under ultrasound irradiation. The produced alkyl radicals and gas were not dependent on oxygen production in vivo and were successfully utilized for SDT and synergistic gas therapy in hypoxic TME. When utilized as a strong US contrast agent, the produced gas bubbles significantly increased the ultrasound contrast to guide cancer therapy. Collectively, the liposome improved the ultrasound imaging and showed enhanced anticancer properties, which might prove beneficial for ultrasound imaging-guided hypoxia-targeted therapy along with deep tissue penetration [84].

### 3.3. Solid Lipid Nanocarriers

Solid lipid NCs (SLNs) are prepared with fatty alcohols, fatty acids, and various glycerides [85]. As compared to other NCs for drug delivery, SLNs provide some advantages as a drug delivery system, including an easier manufacturing process, improved biocompatibility, sustained and controlled release of the cargo molecule, protection of the encapsulated drug from degradation and leaching, and enhanced physical stability [85,86]. In addition, SLNs provide protection to the encapsulated molecules from the harsh environment of the gastrointestinal tract (GIT). A number of in vivo studies have already confirmed that SLNs can ameliorate a drug molecule’s absorption potential and thus bioavailability [87,88,89]. The functions of T-cells can be affected by the redox status of TME by changing the balance between S–S and –SH groups on their surface. This effect on T-cells can be hindered by using drug molecules that can neutralize ROS [90]. Shi et al. [91] pre-treated anti-CD3-coupled fusogenic liposomes with T-cells, which played a role as competitors of T-cell oxidation, which further resulted in the activation of T-cells and the regression of tumors in murine cancer models. In a different study, clodronate-loaded liposomes were used by Fritz et al. [92] to specifically eradicate macrophages in a urethane-induced mouse lung cancer model. The liposomal clodronate treatment reduced the alveolar macrophage populations by over 50% and tumor burden by 50% [92]. A major disadvantage of clodronate liposomes is that they are not specific to macrophage subsets. These NCs target both M1 and M2 macrophages in a similar manner, thus resulting in a comparatively unaffected M1/M2 ratio in TME [92].

TNBC is an aggressive type of breast cancer, that is characterized by the absence of human endothelial receptor 2 (HER2) overexpression, progesterone receptors, and estrogen receptors. These deficiencies in receptor expression make TNBC resistant to numerous currently available targeted therapies, which can result in limited therapeutic options and poor therapeutic outcomes in affected individuals [93]. Rahdari et al. [94] explored a novel therapeutic approach by utilizing C-peptide-conjugated SLNs to target the delivery of paclitaxel (PTX) in the treatment of TNBC. The NCs showed high encapsulation efficiency (around 90%), uniform distribution, appropriate morphology and size, and stability over time. In addition, the NCs showed a prolonged PTX release (lasted around 90 h) and ensured controlled delivery of the drug in the acidic TME. In comparison with the physiological pH (pH 7.4), an enhanced PTX release pattern was also observed in acidic conditions (pH 4.7, 5.5, 6, and 6.5), which mimics TME, and thus enhances drug delivery [94].

### 3.4. Micelles

Micelles are another type of lipid NC formed from the aggregation of amphiphilic molecules [95,96]. Amphiphilic block copolymers are self-assembled into supramolecular structures that contain a hydrophilic outer surface and a hydrophobic core, known as a polymeric micelle [97]. The most commonly utilized polymers for the development of micelles include amphiphilic di-block copolymers, including poly(ethylene glycol) (PEG) and polystyrene, and triblock copolymers, including poloxamers; however, ionic copolymers, including poly(ethylene glycol)-poly(ε-caprolactone)-g-polyethyleneimine) and chitosan-based graft copolymers, are also used [98,99]. A significant improvement has been observed with TME-responsive polymeric micelles in the development of precision therapy to treat cancers [100,101]. TAM-targeting immunotherapy is a promising approach that includes alterations of TME with the immunomodulator imiquimod (R-837) for improved cancer therapy. Unfortunately, extremely limited functions of R837 were observed because of a lack of targeting capacity and its poor water solubility. In order to improve cancer chemo-immunotherapy against breast cancer, Wei et al. [102] prepared two types of targeted polymeric micelles to separately deliver doxorubicin (DOX) and R-837 to tumor cells and TAMs through intravenous and intratumoral injections, respectively. Following the accumulation of these micelles in the tumors, R-837 was released by the immunostimulating micelles; the released R-837 then bound with the toll-like receptor 7 on the lysosomal membrane within the TAM to induce TAM maturation, which eventually resulted in an antitumor immune response and relieved the immunosuppressive effect of TME [102].

Administration of pre-treatments to regulate TME prior to cancer therapy was found to improve efficacy. For instance, pre-treatment with imatinib mesylate, hyaluronidase, and ambroxol might improve the distribution of micelles and buildup in the tumor tissues via reducing vessel density and normalizing tumor vessels [103,104,105]. TME-modulating compounds can easily be incorporated in polymeric micelles; therefore, TME regulation via utilizing polymeric micelles might generate better results in terms of cancer immunotherapy. In a different study, Wang et al. [106] developed a novel photosensitizer carrier through the chemical conjugation of hemoglobin to polymeric micelles to generate triblock copolymers containing poly(ethylene glycol)-block-poly(acrylic acid)-block-polystyrene for photodynamic therapy (PDT). The micelles were found to reverse the hypoxic condition in TME and led to enhanced photocytotoxicity [106].

### 3.5. Nanoemulsions

Nanoemulsion (NE) technology is an NC system composed of proper proportions of water, oil, emulsifier, and co-emulsifier. The particle sizes of NEs range between 10 and 100 nm [107]. NEs have been extensively studied as drug carriers for lipophilic chemotherapeutics because of their controllable drug release, easy preparation, and biodegradability [108,109]. Furthermore, NEs offer a range of advantages, including superior safety and efficacy, good biocompatibility, physicochemical stability, improved bioavailability of drugs, enhanced drug solubility, and prevention of drug inactivation in the GIT [95,110]. In a study, Periasamy et al. [111] developed highly stable *Nigella sativa* containing NEs by using water, polysorbate 80, and ultrasonic emulsification. The developed NEs showed in vitro anti-cancer properties in the MCF-7 breast cancer cell line via triggering apoptosis [111]. In a different study, Natesan et al. [112] developed chitosan-stabilized camptothecin NEs to enhance breast cancer treatment. The developed NEs exhibited tolerable hemolytic potential, prolonged drug release, uniform droplet size distribution, and substantial cytotoxicity against MCF-7 cancer cells and showed lower DNA damage to lymphocytes. In 4T1-breast tumor xenograft BALB/c mouse models, in vivo biodistribution study revealed that camptothecin was passively targeted to breast cancer by chitosan-stabilized camptothecin NEs in comparison with the non-stabilized NE.

### 3.6. Polymer-Based Nanocarriers

Polymer NCs are extensively used in the delivery of anticancer agents owing to their outstanding properties, including functionalized surface, small size, low immunogenicity and inflammation, colloidal stability, biocompatibility, and biodegradability [113]. In a study, Hou et al. [114] developed novel dual-responsive polymeric NCs by utilizing triethylamine as an acid-binding agent, while cysteine derivatives oligomers derived from hexachlorocyclic-triphosphonitrile were polymerized with DOX. The developed NCs have the capacity to target tumor sites through an enhanced permeability and retention effect as well as being able to respond to pH and glutathione for liberating cancer therapeutics. Furthermore, the NCs showed enhanced stability in the blood circulation; however, the response to an acidic pH environment in TME triggered the rapid release of drugs [114].

### 3.7. Dendrimers

Dendrimers are synthetic, hyperbranched, nanosized structures with a spherical shape and their particle sizes range between 1 and 10 nm [115]. Drug molecules can be encapsulated in the dendrimer core by hydrophobic or electrostatic interactions and hydrogen bonds [116]. Dendrimers serve as a promising drug delivery carrier since they can easily be functionalized and offer some exclusive benefits, including antigenicity, reduced immunogenicity, water-solubility, and high stability [95,117,118]. Despite numerous advantages, dendrimers are intrinsically toxic; thus, repeated administration of non-degradable dendrimers can result in toxicity because of their bioaccumulation [119]. Therefore, to decrease such toxicity, various biocompatible dendrimers have been designed, developed, and produced, and surface engineering has been utilized to generate advantageous alterations at the periphery of dendrimers [120].

In a study, Ni et al. [121] developed a novel tumor-targeted and TME-responsive NC based on a core-shell tecto dendrimer for cuproptosis-mediated chemodynamic therapy (CDT) and improved magnetic resonance imaging (MRI). Effective selective tumor-targeting, efficient loading, and TME-responsive release of disulfiram and copper(II) might enhance cuproptosis of cancer cells, improve the intracellular buildup of drug molecules, and increase the synergistic therapeutic effect with CDT, leading to improved MRI and enhanced eradication of tumors [121].

In a different study, Jiang et al. [122] developed a self-assembling dendrimer nanomicelle-based drug delivery system for deeper and more effective tumor penetration through in situ tumor-secreted extracellular vesicles, which is an endogenous-based TME-responsive drug delivery system. After reaching the tumor site, the developed nanomicelles had their payload repackaged via the cells into extracellular vesicles, which were also delivered and internalized through various other cells for transport in relay. The researchers used colorectal and pancreatic cancer-derived xenograft, 2D, and 3D models to demonstrate that the extracellular vesicles produced in situ enhanced intercellular delivery and propagation of cargos from cell to cell and deeper penetration within the tumor [122].

### 3.8. Nanoclays

**Figure 3:**
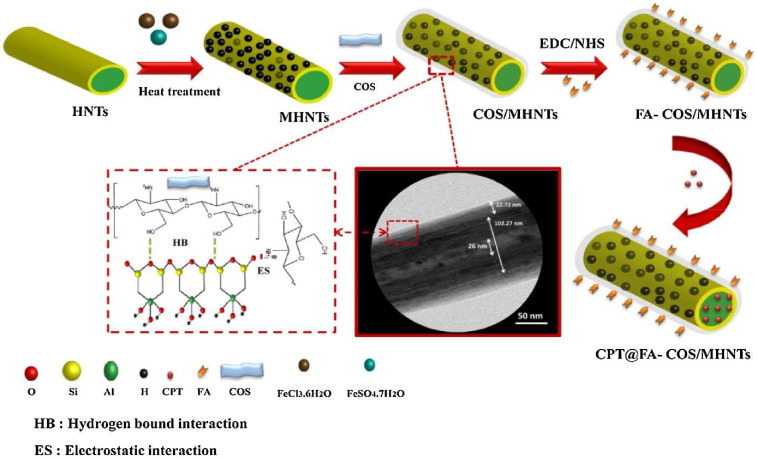
Synthesis of camptothecin (CPT)-loaded folic acid-conjugated chitosan oligosaccharides assembled magnetic halloysite nanotubes (FA-COS/MHNTs). Reproduced with permission from Elsevier, Reference [124]. Abbreviations: FA, folic acid; COS, chitosan oligosaccharide; CPT, camptothecin; MHNTs, magnetic halloysite nanotubes; EDC/NHS, N-(3-dimethylaminopropyl)-N/-ethylcarbodiimide/N-hydroxysuccinimide.

Nanoclay is a layered mineral silicate nanostructure with a particle size of less than 100 nm. There is a growing interest in its use in cancer diagnosis and treatment because of its unique advantages, including strong adsorption capacity, high specific surface area, adjustable morphology, strong cation exchangeability, good biocompatibility, and low price [123]. Camptothecin is a strong topoisomerase I inhibitor which is used to treat metastatic colorectal cancer. The major drawbacks of camptothecin include toxicity to non-tumor tissues and poor water solubility. In order to overcome these drawbacks, halloysite nanotubes have been developed as a delivery system for the delivery of camptothecin. As unique tubular nanoclays, Dramou et al. [124] developed a unique delivery system based on halloysite nanotubes, which were functionalized with folic acid and modified with chitosan oligosaccharides in order to effectively deliver camptothecin (Figure 3). The CPT-loaded NCs exhibited a powerful cell growth inhibitory effect against human colon carcinoma cells. Interestingly, the release rate of camptothecin at the acid pH (pH 5) of TME was higher than at pH 6.8 as well as pH 7.4. Moreover, the developed NCs selectively targeted cancer cells owing to their improved cell uptake mediated by chitosan oligosaccharides and folic acid [124].

In another study, Zhang et al. [125] developed a typical nanoclay container based on methoxy-intercalated kaolinite for targeted DOX delivery to enhance DOX delivery to the cancer tissues while having reduced side-effects in the treatment of thyroid cancer. An increased level of DOX was released from the targeted delivery system in the acidic TME of cancer tissues as compared to macrophages, owing to the bigger size and enhanced level of acidic metabolic products in cancer tissues in comparison with normal tissues. The developed DOX-loaded methoxy-intercalated kaolinite showed dose-dependent therapeutic effects in vitro and played a role as a strong targeted therapeutic drug delivery system [125].

### 3.9. Nanocrystals

Nanocrystals are a distinct class of NCs that are composed of pure drug particles in the form of crystals [126]. Formulations based on nanocrystals have already salvaged a number of poorly soluble drugs. The particular advantages of nanocrystals include enhanced drug-loading efficiency, greater structural stability, steady dissolution rates, and high surface-area-to-volume ratio. These advantages are mainly linked with the composition of nanocrystals, as they consist entirely of drug molecules, which further eliminates the additional requirement of a carrier and results in significant therapeutic levels at lower doses [127]. In a study, Zhou et al. [128] developed catalase nanocrystals as an in situ oxygen-generating system to enhance PDT efficiency and reduce tumor hypoxia in solid tumors. The developed nanocrystals enabled prolonged endogenous decomposition of hydrogen peroxide to supply oxygen continuously for the prolonged relief of tumor hypoxia. Thus, the nanocrystals eradicated the hypoxia and enhanced the effectiveness of PDT [128].

### 3.10. Exosomes

Exosomes are naturally occurring NCs that are released by various cell types and are also present in several types of body fluids [129]. The features of exosomes include smaller size, lower immunogenicity compared to artificial drug carriers, enhanced ability to penetrate through a range of biological barriers, high stability, natural targeting capacity, and potential derivation from patients’ cells [130]. In addition, exosomes offer a stable environment for drug molecules and their targeting capacity can be improved further by adding functional drugs or conditioning parental cells [131]. In a study, Kim et al. [132] loaded PTX into exosomes and modified it with aminoethylanisamide-polyethylene glycol in order to target sigma receptors of overexpressed lung cancer cells. The PTX-loaded exosomes showed greater loading capacity, high capacity to build up in cancer cells, and enhanced therapeutic outcomes. Exosomes loaded with anticancer drugs can also control TME. Wang et al. [133] demonstrated that PTX can be loaded into M1 macrophage-derived exosomes. The developed exosomes enhanced inflammation and mediated a pro-inflammatory environment, which further induced apoptosis, increased expression of caspase-3, and eventually enhanced the anticancer property of PTX [133].

### 3.11. Carbon Nanotubes (CNTs)

CNTs are promising NCs composed of graphene sheets that are seamlessly rolled up to form hollow cylinders [134]. CNTs can serve as a multifunctional NC that combines thermal ablation [135], bioimaging [136], and tumor-targeted drug delivery [135]. Furthermore, CNTs are treated with various strong acids in order to functionalize their surfaces with carboxylic acid groups [135]. Various anticancer drugs can be loaded inside the CNTs or can be attached to their surfaces [135]. Cancer cells uptake CNTs through endocytosis and passive diffusion in order to deliver drug molecules selectively to the mitochondria [137], nucleus [138], and cytoplasm [139] of target cells to treat cancers [135]. In a study, García-Hevia et al. [140] explored the efficacy of oxidized multi-walled CNTs containing PTX in suppressing metastatic growth via enhancing cytotoxic, anti-migratory, and anti-proliferative properties in both TME and cancer cells. The developed CNTs reduced malignant melanoma lung metastases by over 80%.

### 3.12. Selenium Nanocarriers

Selenium NCs have already demonstrated their strong antiproliferative actions against various cancer types, including lung and breast cancers [141,142]. In a study, Ferro et al. [143] developed bovine serum albumin-stabilized selenium NCs. In combination with a KRAS*_wt_* nanovaccine, the developed NCs exerted antitumor functions and significantly regulated tumor growth in mice bearing EO771 cells derived from spontaneous breast cancer. The combined therapy also increased B-cell infiltration in TME, decreased the levels of Treg cells, and elevated both intratumoral and systemic levels of activated CD8^+^ T-cells (CTLs). Collectively, these findings suggest the potential of bovine serum albumin-stabilized selenium NCs in cancer treatment.

### 3.13. Magnetic Nanocarriers

Magnetic NCs are nano-platforms that involve various moieties based on magnetic NCs for therapeutic purposes. The particle size of magnetic NCs ranges between 1 and 100 nm. The movement of magnetic NCs can be precisely regulated under an external magnetic field. Moreover, magnetic NCs can be externally operated, which allows a non-invasive technique for remote-controlled therapies [144]. In a study, Rao et al. [145] demonstrated that magnetic NCs can be coated with membranes derived from genetically engineered cells that overexpressed an SIRPα variant for CD47 binding and exhibited a 50,000-fold augmented binding affinity. The developed magnetic NCs effectively accumulated in TME under external magnetic field guidance and selectively blocked the macrophage-suppressing CD47–SIRPα binding between macrophages and tumor cells. In addition, this magnetic navigation method with the magnetic NCs also reduced the risk of severe side-effects via improved tumor targeting [145].

### 3.14. Quantum Dots (QDs)

QDs are a newer type of NCs composed of small inorganic semiconductor nanocrystals with a particle size between 1 and 10 nm. There is growing interest in QDs as a targeted drug delivery system because of their modifiable and unique physicochemical properties. In general, QDs contain a semiconductor core, which is coated with a shell to alter its chemical and physical properties and enhance solubility [95]. In a study, Li et al. [146] developed three organic polymers to alter black phosphorus (BP) QDs. They first simultaneously grafted ultra-small BPQDs with three different functional pyrene-ended polymer brushes, including ROS-sensitive polypropylene sulfide (PPS), polyacrylic acid (PAA) along with an Ag^+^ ions-trapping activity, and hydrophilic PEG, which they termed BPQD@PAA/PEG/PPS. The self-assembly nature of the developed QD resulted in Ag^+^ embedding in the PPS shell layer, exploiting PPS shell’s hydrophobicity for effective protection of Ag^+^ and the attainment of Ag^+^-coupled BP vesicles. After intravenous administration of these vesicles in 4T1-bearing mouse models, the Ag^+^-coupled BP vesicle QDs showed selective accumulation within the tumor over time. Following 660 nm irradiation, this triggered substantial necrosis and apoptosis of tumor cells. Moreover, TME exhibited increased concentrations of pro-inflammatory factors, which possibly improve anti-cancer immunotherapy [146].

### 3.15. Black Phosphorus Nanosheets (BPNSs)

BPNSs are a new class of NCs composed of two-dimensional layered materials, which have been extensively studied in cancer treatment because of their low toxicity, optical properties, and excellent electrical conductivity [147]. BPNSs have also been utilized in preclinical studies as a photothermal agent in the treatment of tumors [148]. Kumar et al. [149] developed near-infrared-responsive niosome-coated BPNSs to provide PDT to treat cancers. The developed niosome-coated BPNSs showed a very high drug loading efficiency (>90%) when loaded with DOX and indocyanine green. The BPNSs also exhibited effective tumor cell uptake as well as tumor cell death, and outstanding cytocompatibility in the dark. In order to simulate TME, the developed BPNSs were studied against the 3D tumor spheroids, where the BPNSs showed uptake by the tumors and caused death of cancer cells. Moreover, lasers can also be used to control the therapeutic effectiveness of the developed BPNSs, which show light-responsive behavior in vitro, to eradicate cancer cells [149].

### 3.16. Hybrid Nanogels

Nanogels are promising NCs that can be used to enhance the safety and efficacy of various anticancer drugs. Hybrid nanogels were found to have the capacity to react with both external and internal stimuli [150]. To date, a range of organic and inorganic nanomaterials have been incorporated into nanogels to develop hybrid, highly responsive, and multipurpose NCs. As compared to simple NCs, hybrid nanogels offer more fluidity and softness, which is useful as a drug delivery system because it makes it more convenient for the cells to uptake the nanocomposites [151,152]. Intratumoral CTLs are important for efficient cancer immunotherapy; however, an immunosuppressive TME can play a role in dysfunctions and inadequate infiltration [153]. In a study, Tian et al. [154] developed self-degradable nanogels (PMI nanogels) containing two immune modulators, including metformin and imiquimod. The developed PMI nanogels showed TME-responsive drug release. In addition, the PMI nanogels remodeled TME by downregulating PD-L1 expression, repolarizing M2-like tumor-associated macrophages, and mediating dendritic cell maturation. Collectively, the nanogels remodeled immunosuppressive TME and effectively mediated the activation and infiltration of CTLs [154].

## 4. Preclinical and Clinical Studies of Nanocarrier-Mediated Drug Delivery to Remodel the Tumor Microenvironment for Cancer Therapy

Numerous NCs have already demonstrated their potential by inducing distinct anticancer mechanisms by delivering a range of anticancer drugs in various preclinical studies (Table 1). Some of the NCs have already been approved by the US Food and Drug Administration (FDA) and some have entered different clinical phases (Table 2).

## 5. Current Challenges and Future Directions

It is challenging to achieve a good therapeutic effect by using a single therapy owing to the numerous immunosuppressive mechanisms present in tumors. Multifunctional NCs and their controlled release system can efficiently suppress several immune signaling pathways; thus, they can provide effective cancer immunotherapy. Numerous NC-based formulations have been used to modulate TME because of their potential to improve the effectiveness of cancer therapies [16]. Nonetheless, there are several challenges that need to be overcome in the translation of NC-based anticancer therapies into clinical practice. Limited knowledge regarding the tumorigenesis-associated immune network largely limits the use of NCs in cancer immunotherapy. Variation in the therapeutic effects is another issue with the use of NCs. This phenomenon is mainly due to the heterogeneity of the structures of different tumors because of the differences in their vasculatures; thus, responses of different tumor types to the same therapy can be different. There are also some concerns regarding the possible risks of NCs, for example, the potential immunogenicity caused by NCs themselves, and currently there is a lack of robust toxicity assays for NCs [17]. Thus, more studies are required to optimize the ligands, shapes, sizes, and various other properties of NCs, and the potential risks associated with NCs also need to be carefully evaluated before their translation into clinical practice.

A large number of studies have reported the potential of NCs and this number is increasing every year; however, comparatively little translation has occurred from the bench to the bedside. Interactions between NCs and the complex immunological system, including the mononuclear phagocyte system, result in the rapid clearance of most of the NCs, which is a major challenge for the clinical translation of NC-based drug-delivery systems [162]. Various studies are utilizing NC surface modification to unravel such complex interactions. A range of surface functionalization approaches, including surface protein addition [163,164], PEGylation [165], pH or ion sensitivity [166,167,168], and overall charge [166], have been designed and developed to increase the retention time of NCs and delineate the physiological target within the body.

Despite promising outcomes in numerous preclinical animal studies, NC-based cancer therapies, due to lack of efficacy, often fail in clinical studies during phase II and phase III trials. This failure rate can perhaps be decreased by setting rigorous criteria for both testing as well as quality control during the design and development steps, and by executing carefully planned preclinical studies in pertinent animal models [169].

Indeed, researchers need to carefully design and carry out their studies and report the study outcomes with transparency and accuracy for successful translation of NC-based cancer therapies from preclinical testing to human use. In addition, before animal studies, researchers should consider the usage of a chorioallantoic membrane model or organ-on-a-chip to evaluate NC-based cancer therapies [170]. These study models may elucidate which NCs are most likely to be successful in rodent models, and eventually in human subjects. It should be noted that studies conducted in less complex species, including mouse models, ought to be designed and developed along with transition to those of a higher degree of complexity. Therefore, researchers should consider utilizing study models that represent various factors, including the role that inflammation plays in tumorigenesis [171] and the age of a typical cancer patient [172]. At present, most animal studies use young rats or mice; however, it is older humans who are mainly affected by cancer. Thus, the use of older rodents for preclinical studies might ameliorate the link between preclinical and clinical study results. Furthermore, clinicians should also be involved at every stage of the design and development process, since their knowledge and experience might facilitate the successful transition of NC-based cancer therapies to human use [169].

Another major challenge is the development of a manufacturing method that enables the transfer of laboratory-based NC production to industrial-scale production with proper quality control and characterization techniques. Various factors are linked with the scale-up of NC-based cancer therapy from bench to human use. Such factors include the toxicological properties linked with the shape and size of NCs, the nature of the material and its generally regarded as safe (GRAS) status, in vivo biodegradability of NCs, and balancing of multicomponent systems at large scale [173]. Both patients and clinicians need to consider a range of factors prior to the selection of NC-based therapies, including cost, method of NC development, solvent, and the acceptability of the finished products. Sometimes the desired properties of NCs are lost during the scale-up of a laboratory method [174]. For instance, in a study, Colombo et al. [175] used an emulsion method to scale up NCs. The researchers revealed that an increase in agitation time and impeller speed resulted in decreased particle size, although there was no alteration in entrapment efficiency. From the scale-up point of view, the correct selection of the NC production method is crucial in saving time during pilot batch production [173].

## 6. Conclusions

There is a growing interest in NC-based targeted therapies in terms of cancer treatment. NC-based drug delivery systems provide enhanced pharmacokinetic profiles of anticancer agents, which can substantially improve immune responses, avoid cytokine storm owing to immune hyperactivation, and reduce side-effects. Various NC-based formulations have already demonstrated their potential in TME modulation. NCs that elevate blood perfusion and disturb the tumor vasculature and/or ECM can enhance the penetration as well as the intracellular transfer of anticancer drugs. NCs that modulate Treg cells, TAMs, CAFs, and DCs have the capacity to modify the populations and functions of immune cells in TME. Moreover, NC-based drug delivery systems can also be combined with various other targeting approaches. Indeed, multifunctional NCs have a great future in the delivery of anticancer agents to modulate TME. However, in order to harness the full potential of NCs in tumor treatment, there are several factors that need to be carefully studied, including optimization of drug loading into NCs, NC-associated immunogenicity, and biocompatibility. Moreover, emerging systems including biohybrid NCs can be further explored in terms of TME-targeting, as these NCs have great potential in cancer immunotherapy, chemotherapy, and combined therapy. Biohybrid NCs have the capacity to provide unique biofunctionalities supplied by integrated cells and preserve the physicochemical features of the synthetic materials. Artificial intelligence can also help in biomarker detection and in bridging the gap, predicting NC interactions of the targeted drug, and assessing drug efficacy. A combination of NCs and photodynamic therapy might also be considered, which can provide improved therapeutic outcomes and lower side-effects because of the guided TME accumulation.

## Figures and Tables

**Table 1:** Preclinical studies of nanocarrier-mediated drug delivery to remodel the tumor microenvironment for cancer therapy.

Nanocarriers (NCs)	Loaded Compounds or Drugs	Average Particle Size	Targeted Disease	Study Outcome	References
Mesoporous silica NCs	Doxorubicin (DOX)	99.8 ± 6.3 nm	Cancer	Showed on-demand rapid release in the tumor microenvironment (TME), which might have significantly contributed to the selective eradication of tumor cells and saving of the normal cells	[155]
Hollow mesoporous silica NCs	Interleukin-2, DOX, and all-trans retinoic acid	~180 nm	Cancer	NCs-mediated combination therapy mediated benign regulation on TME by mediating secretion of various cytokines, including IL-12 and IFN-γ, activating natural killer cells and T-lymphocytes, and down-regulation of immunosuppressive cytokines (TGF-β and IL-10) and immunosuppressive myeloid-derived suppressor cells	[75]
Liposomal NCs	anti-IL6R antibodies	∼100 nm	Breast cancer	Moderated TME and suppressed IL6R-Stat3 signaling cascade, as characterized through the decreased expression of various genes encoding CD206, MMP-9, VEGFA, Sox2, and Stat3 in breast tissues	[82]
Liposomal NCs	2,2′-azobis [2-(2-imidazolin-2-yl)propane]dihydrochloride (AIPH)	∼100 nm	Cancer	AIPH-loaded liposome concurrently produced gas bubbles as well as a high level of reactive oxygen species (ROS) under ultrasound irradiation. The produced alkyl radicals and gas were not dependent on oxygen production in vivo and were successfully utilized for sonodynamic therapy (SDT) and synergistic gas therapy in a hypoxic TME	[84]
Solid lipid NCs	Paclitaxel (PTX)	240 nm	Breast cancer	The NCs showed high encapsulation efficiency (around 90%), uniform distribution, appropriate morphology and size, and stability over time. The NCs showed a prolonged PTX release (lasting around 90 h) and ensured controlled delivery of the drug in the acidic TME. In comparison with the physiological pH (pH 7.4), an enhanced PTX release pattern was also observed in acidic conditions (pH 4.7, 5.5, 6, and 6.5), which mimics TME, thus enhancing drug delivery	[94]
Polymeric micelles	DOX and immune adjuvant R-837	117.6 nm and 110.1 nm	Breast cancer	Two types of targeted polymeric micelles to separately deliver DOX and R-837 to tumor cells and TAMs through intravenous and intratumoral injections. Following accumulation of the micelles in the tumors, R-837 was released by the immunostimulating micelles. The released R-837 then bound with the toll-like receptor 7 on the lysosomal membrane within the TAM to induce TAM maturation, which eventually resulted in an antitumor immune response and relieved the immunosuppressive effect of TME	[102]
Polymeric NCs	DOX	119 nm	Non-small cell lung cancer, colorectal cancer, gastric cancer	The NCs showed enhanced stability in blood circulation; however, the response to the acidic pH environment in TME triggered the rapid release of drugs	[114]
Dendrimers	Tirapazamine	4.72 ± 0.80 nm	Breast cancer	The developed TME-responsive NCs effectively and selectively eliminated tumors by the synergistic effect of CDT and chemotherapy, which were found to be safe and effective as a tumor therapy	[156]
Nanoclays	Camptothecin (CPT)	421.53 ± 29.263	Colorectal cancer	The CPT-loaded NCs exhibited a powerful cell growth inhibitory effect against human colon carcinoma cells. Interestingly, the release rate of camptothecin at the acid pH (pH 5) of TME was higher than pH 6.8 as well as pH 7.4	[124]
Nanoclays	DOX	150 to 200 nm	Thyroid cancer	An increased level of DOX was released from the targeted delivery system in the acidic TME of cancer tissues as compared to macrophages, owing to the bigger size and enhanced level of acidic metabolic products in cancer tissues in comparison with the normal tissues	[125]
Nanocrystals	Methylene blue	900 nm	Hypoxia of cancer cells	The developed nanocrystals enabled prolonged endogenous decomposition of hydrogen peroxide to supply oxygen continuously for the prolonged relief of tumor hypoxia	[128]
Exosomes	PTX	172.8 nm	Breast cancer	PTX was loaded into M1 macrophage-derived exosomes. The developed exosomes enhanced inflammation and mediated a pro-inflammatory environment, which further induced apoptosis, increased expression of caspase-3, and eventually enhanced the anticancer property of PTX	[133]
Carbon nanotubes	PTX	122 nm	Metastatic cancer	Carbon nanotubes containing PTX suppressed metastatic growth via enhancing cytotoxic, anti-migratory, and anti-proliferative properties in both TME and cancer cells	[140]
Magnetic NCs	Genetically engineered cell membrane shell	~100 nm	Melanoma and breast cancer	The developed magnetic NCs effectively accumulated in TME under external magnetic field guidance and selectively blocked the macrophage-suppressing CD47–SIRPα binding between macrophages and tumor cells	[145]
Black phosphorus nanosheets (BPNSs)	DOX and indocyanine green	160 nm	Lung cancer	In order to simulate TME, the developed BPNSs were studied against 3D tumor spheroids, where the BPNSs showed uptake by the tumors and caused death of cancer cells	[149]

**Table 2:** Clinical studies of nanocarrier-mediated drug delivery to remodel tumor microenvironment for cancer therapy.

Nanocarriers (NCs)	Loaded Drugs or Compounds	Cancer Type	Clinical Stage/FDA Approval	References
Gold NCs	Small interfering RNA	Glioblastoma	Phase 0	[157]
Polymeric micelles	Paclitaxel	Advanced, refractory malignancies	Phase 1	[158]
NCs	Albumin-bound paclitaxel	Metastatic breast cancer	FDA approved	[159]
Liposomal NCs	Doxorubicin	Multiple myeloma, metastatic breast cancer and ovarian cancer	FDA approved	[160]
Polymeric micelles	Paclitaxel	Metastatic breast cancer and non-small-cell lung cancer	Phase 2	[161]

## Data Availability

The data presented in this study are contained within this article.
